# Colloidal Crystallization of Virus‐Like Particles with Polycations

**DOI:** 10.1002/smll.202503579

**Published:** 2025-07-02

**Authors:** Bettina Tran, Timothy G. Keys, Milad Radiom, Stefan Salentinig

**Affiliations:** ^1^ Department of Chemistry Food Research and Innovation Center National Center of Competence in Research Bio‐inspired Materials University of Fribourg Chemin du Musée 9 Fribourg 1700 Switzerland; ^2^ Department of Health Sciences and Technology Eidgenössische Technische Hochschule Zürich Schmelzbergstrasse 9 Zürich 8092 Switzerland

**Keywords:** colloidal crystals, nanostructured material, self‐assembly, small‐angle X‐ray scattering (SAXS), virus‐like particles

## Abstract

Virus‐like particles (VLPs) are protein nanocages capable of encapsulating or attaching guest molecules. Unlike viruses, they do not replicate in cells, making them promising candidates for advanced biomaterial design, particularly for biomedical applications such as drug delivery. However, the mechanisms governing VLPs self‐assembly into highly ordered suprastructures with enhanced functionality remain largely unexplored. This study investigates the development of pH‐responsive biomaterials using the icosahedral *Acinetobacter* phage coat protein AP205 VLPs, which has a diameter of ≈28 nm. Small‐angle X‐ray scattering, dynamic light scattering, and zeta‐potential measurements reveal that AP205 VLPs self‐assemble with the polycation poly[2‐(methacryloyloxy)ethyl] trimethylammonium chloride (pMETAC) into highly ordered suprastructures. The structural organization is strongly influenced by composition, pH, and ionic strength. The findings provide insights into the directional interactions governing VLPs self‐assembly with polycations and can guide the design of advanced, tunable VLP‐based biomaterials.

## Graphical abstract

This study demonstrates the formation of colloidal crystals from virus‐like particles combined with a short‐chain polycation. It emphasizes the roles of surface charge, roughness, and environmental pH on the geometrical order of the suprastructures. The knowledge guides the creation of advanced pH‐responsive colloidal crystals for biomedical applications such as drug delivery.

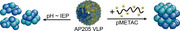

## 1 Introduction

Effective drug delivery systems are crucial for achieving targeted, controlled, and efficient therapeutic delivery while enhancing stability or bioavailability.^[^ ^1^ ^]^ Drug encapsulation within nanostructured materials is a widely researched approach to boosting overall efficacy.^[^ ^2^, ^3^, ^4^ ^]^ These materials can be functionalized to respond to specific triggers for drug release, such as the pH value of infected tissues or tumors.^[^ ^5^ ^]^

The self‐assembly of protein nanocages, including non‐enveloped viruses, bacteriophages, virus‐like particles (VLPs), and protein structures like ferritin, has been studied for advanced material design applications.^[^ ^6^, ^7^, ^8^, ^9^, ^10^, ^11^ ^]^ They allow encapsulating or attaching drug molecules and tailoring surface modification for targeted delivery.^[^ ^12^, ^13^, ^14^, ^15^, ^16^ ^]^ VLPs are a class of protein nanocages that are colloidally and structurally similar to wild viruses but lack genomes for replication. They are safe and non‐infectious for biomedical applications.^[^ ^17^, ^18^ ^]^ The coat proteins that assemble into VLPs can be derived from the coat protein units of mammalian viruses, bacteriophages, and plant viruses.^[^ ^19^ ^]^ VLPs are generally robust structures, allowing for the chemical conjugation of various molecules (e.g., targeting molecules, antigenic compounds) and/or encapsidation of diverse compounds (e.g., drug molecules, fluorescent molecules, and metal nanoparticles).^[^ ^20^, ^21^, ^22^ ^]^

Their precise size, shape, and surface chemistry of protein nanocages make them ideal building blocks for designing advanced functional materials. For instance, rod‐like phages, such as fd or M13 bacteriophages, were used to form hexagonally packed structures or star‐like assemblies.^[^ ^6^, ^8^ ^]^ Isotropic nanocages such as P22 VLP, cowpea chlorotic mottle virus, and ferritin have been arranged in cubic or hexagonal lattices.^[^ ^23^, ^24^ ^]^ Structurally anisotropic phages have been researched as delivery vehicles in tumor targeting because they can traverse biological barriers more easily.^[^ ^14^, ^25^ ^]^ However, their self‐assembly into programmable, responsive structures has not been widely studied.

Interactions among protein nanocages and their stability against aggregation in suspension were discovered to be highly pH‐dependent, with reversible aggregation into micron‐sized aggregates occurring at a pH close to their isoelectric point.^[^ ^26^, ^27^ ^]^ The stimuli‐responsive aggregation and de‐aggregation of phages could enable the targeted delivery of high concentrations of phages to specific areas in the body. Our group has recently discovered pH‐responsive hexagonally structured supraparticles, formed from the self‐assembly of the 27 nm diameter *Escherichia coli* (*E.coli*) bacteria virus *Qubevirus durum* (Qbeta) with the polycation poly [2‐(methacryloyloxy)ethyl] trimethylammonium chloride (pMETAC) of defined chain length.^[^ ^10^ ^]^ This self‐assembly is primarily driven by the specific adsorption of pMETAC onto the virus capsid surface, followed by anisotropic electrostatic interactions. Notably, the phage remains biologically active within the assembled structures. The supraparticles could be disassembled by external triggers, such as changes in the pH or the salt concentration in the suspension, to release the fully active phages.

A significant gap remains in understanding the specific properties of viral capsids and the inter‐capsid interactions that govern their self‐assembly into ordered structures, as opposed to random aggregation. Electrostatic attractions among nanocages can be tailored by adding charged components, such as polypeptides, dendrimers, nanoparticles, metal ions, or even co‐assembly with a different nanocage.^[^ ^11^, ^24^, ^28^ ^]^ The nanocages can further be precision bioengineered to obtain selected functional groups on their surface for inter‐particle interactions.^[^ ^29^ ^]^ In addition to electrostatic forces, hydrophobic interactions can modify the resulting geometrical orientation of the aggregates.^[^ ^27^, ^30^, ^31^ ^]^ Aromatic stacking or coil‐coil interaction through surface modification by covalently attached molecules can guide assembly and diminish the dependence of the resulting structure on the nanocage's initial shape.^[^ ^7^, ^32^ ^]^ This is also evident in cases where electrostatic templating of nanocages occurs before crosslinking, with the binding depending on the specific protein sequence.^[^ ^33^, ^34^ ^]^

This work fills some of these knowledge gaps and reports the creation of suprastructured biomaterials from the *Acinetobacter* phage coat protein (AP205) VLP with pMETAC. AP205 VLP is formed from 90 copies of AP205 dimers, assembling into an icosahedral geometry of a diameter of ≈28 nm.^[^ ^12^, ^35^ ^]^ The VLP has been studied and optimized for immunization and vaccine delivery.^[^ ^12^, ^36^, ^37^, ^38^, ^39^, ^40^ ^]^ In particular, because AP205's N‐ and C‐terminal groups of the coat proteins protrude from the capsid surface, its purification and additional antigen presentation are facilitated.^[^ ^35^, ^41^ ^]^ This unique feature of AP205 VLP, compared to other commonly used VLPs such as MS2, combined with the potential of encapsulating guest molecules, makes AP205 VLP an ideal building block for advanced structured biomaterials for delivery applications. Furthermore, the AP205 VLP is similar in size to Qbeta, has a smoother surface, and features a more uniform charge distribution.^[^ ^41^, ^42^ ^]^ These distinctions offer valuable insights into how those properties, especially regarding surface roughness, distributions of surface charge, and hydrophobic groups, affect the protein nanocage self‐assembly to form suprastructures. Small‐angle X‐ray scattering (SAXS), dynamic light scattering (DLS), and zeta potential are applied to characterize the AP205 VLP suprastructure formation in the presence and absence of pMETAC, depending on ionic strength and pH in suspension. These findings advance the understanding of VLP self‐assembly with polycations into advanced biomaterials.

## 2 Result and Discussion

### 2.1 Characterization of AP205 VLP in PBS

**Figure 1:**
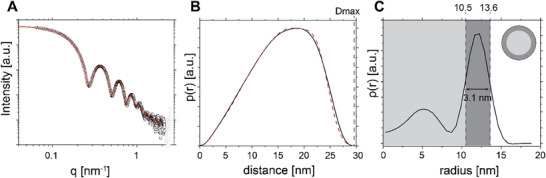
A) Experimental SAXS data of 1 wt% AP205 VLP in PBS at pH 7.4 (symbols) and the corresponding best possible fit to the experimental data (red line) from the GIFT analysis. The corresponding structure factor parameters are presented in Table S1 (Supporting Information). B) The pair‐distance distribution function (p(r), black line), obtained from the GIFT analysis with the corresponding best possible fit from deconvolution (dashed red line). Note that r in this plot represents the characteristic distance. C) The resulting ρ(r) assumes radial symmetry with r being the radius. The width of the second maximum ("shell") was determined by the points of inflection, to focus on the highest excess electron density. The inset represents the electron density distribution of the core‐shell structure, according to the ρ(r). Residuals from the GIFT fitting and the determination of the points of inflection in ρ(r) are provided in Figure S1 (Supporting Information).

The size and shape of the AP205 VLP in phosphate buffer saline (PBS, 1x, 137 mM, 2.7 mM KCl, 10.1 mM Na_2_HPO_4_ 2H_2_O, and 1.8 mM KH_2_PO_4_) at pH 7.4 were analyzed with SAXS, DLS, and zeta potential measurements. The SAXS curve of AP205 VLP at a concentration of ≈1 wt% in **Figure** **1**A shows the characteristic features of spherical nano‐sized particles. The overall scattering of the relatively monodisperse nanoparticle can be observed at low magnitude of the scattering vector (*q*) < 0.1 nm^−1^, followed by a Guinier region and the characteristic maxima and minima from the form factor scattering, *P(q)* of a spherical object.

**Figure 2:**
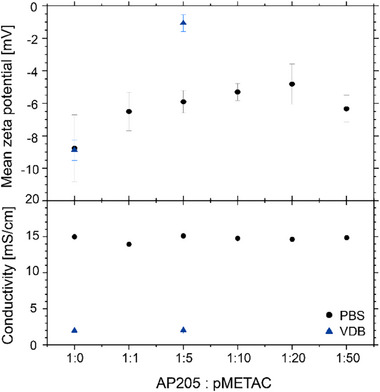
Zeta potential and conductivity of AP205 VLP at different weight ratios of pMETAC in PBS. The samples at 1:0 and 1:5 were further analyzed at lower ionic strength VDB.

To further study the size, shape, and morphology of AP205 VLP, the SAXS data were analyzed with the generalized indirect Fourier transformation (GIFT) method (for details, see supporting information)^[^ ^43^, ^44^ ^]^ The measured zeta potential in PBS of −8.8 ± 2.1 mV (see **Figure** **2**) was used to approximate the effective particle charge for the structure factor, *S(q)*, for the fitting procedure. Thes structure factor, *S(q)*, parameters are summarized in Table S1 (Supporting Information). The effective interaction radius and volume fraction were varied to obtain the best possible fit to the experimental data, resulting in 18.3 ± 0.5 nm and 1.2 ± 0.1%. The volume fraction shows good agreement with the experimental mass fraction of ≈1 wt%. The resulting pair distance distribution function, *p(r)*, shows a maximum distance ≈30 nm, obtained from *p(r)* = 0. This aligns reasonably well with the radius from the *S(q)* model, see Figure 1B. Its shape is consistent with a quasi‐spherical object.

The excess electron density distribution, *ρ(r)* was calculated from the *p(r)* using a convolution square‐root operation to further analyze the morphology of the VLPs.^[^ ^26^ ^]^ The radial excess electron density profile is shown in Figure 1C. The inflection points in the excess electron density profile between the core and shell regions, for 9 < *r* < 16 nm, provide an estimated radius of the virus core and shell thickness, depending on the highest electron density in the shell. The overall particle radius of the AP205 VLP is ≈15 nm, which agrees with the *p(r)* analysis with D_max_/2 around 15 nm. We further used the point of inflections in the *ρ(r)* profile of the shell to estimate the the core and core + shell radius. This results in a shell thickness of around 3.1 nm. This procedure minimizes the contribution of the smeared lower electron densities on the inner and outer surface, e.g., from protruding peptides, and deviations from spherical symmetry. The resulting shell thickness and radius are in reasonable agreement with previous studies, which report a radius of ≈14 nm.^[^ ^12^, ^35^ ^]^ The size obtained from SAXS analysis also agrees with the results from DLS, demonstrating a hydrodynamic radius (R_H_) of 16.7 ± 0.1 nm from the Contin fit with intensity weighting (see Figures S2 and S3, Supporting Information).

### 2.2 The Influence of the Buffer Ionic Strength

AP205 VLPs at reduced ionic strength were characterized in a buffer containing only 5 mM total phosphate concentration and 10 mM NaCl (details on the composition can be found in the SI). This buffer is further referred to as the virus dilution buffer (VDB). The reduced buffer capacity allows the pH to be adjusted by adding a reasonable amount of HCl and NaOH.

The SAXS curves of AP205 VLPs in VDB at pH = 7.0 exhibit the same features as AP205 VLPs in PBS, showing no significant change of the VLPs under the reduced ionic strength (see Figure S4, Supporting Information). DLS of AP205 VLPs in VDB showed a hydrodynamic radius of R_H_ = 16.9 ± 0.2 nm from the Contin fit (see Figures S2 and S3, Supporting Information), also in agreement with the size obtained for AP205 VLPs in PBS. SAXS and DLS confirm that the AP205 VLPs retains its original shape in PBS and VDB under experimental conditions.

**Figure 3:**
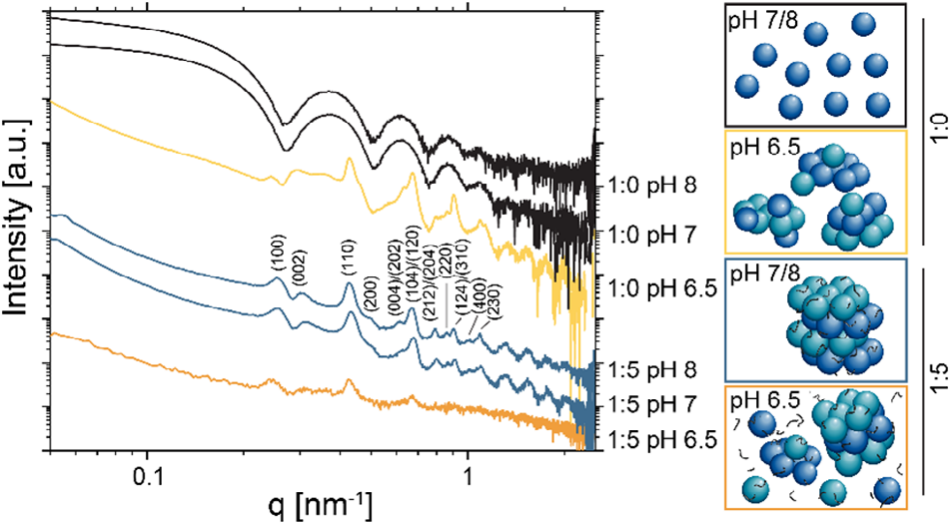
SAXS of AP205 VLP:pMETAC = 1:0 and 1:5 in VDB at different pH values. Graphical representations of the suprastructure are presented as inserts, framed with the color of the corresponding SAXS curve. For the SAXS patterns at AP205 VLP:pMETAC = 1:5 at pH 7.0 and 8.0, the peaks are assigned with their corresponding Miller indices (hkl), which match the hexagonal closed‐packed (hcp) structure. The curves were vertically shifted for clarity.

pH‐dependent changes of AP205 VLPs in VDB were studied using electrophoretic mobility and SAXS measurements. The SAXS curves of AP205 VLPs at pH 7.0 and 8.0 in **Figure** **3** (black lines) represent quasi‐spherical particles, indicating suspensions of AP205 VLPs. However, the curve at pH 6.5 (yellow line) shows an upturn at low q emerging from attractive inter‐particle interactions (aggregation) and Bragg reflections, indicating some ordered structure. To further analyze the potential electrostatic nature of this pH‐triggered AP205 VLP aggregation process, pH‐dependent zeta potential analysis was performed at pH 3.0, 4.0, 5.0, 6.0, 6.3, 6.5, 7.0, and 8.0. The zeta potential decreases from ≈15 to −9 mV with increasing pH values and crosses 0 mV between pH 6.0 and 6.3, marking the isoelectric point (IEP) of AP205 VLP (see Figure S5, Supporting Information). Hence, electrostatic repulsion at pH 6.5 is compromised, which triggers the aggregation process into partially geometrically ordered structures that show multiple Bragg reflections in SAXS. However, the detailed structural assignment for this sample was not feasible in this study.

### 2.3 Colloidal Crystals from AP205 with pMETAC

To create colloidal crystals of AP205 in PBS at pH values > 6.5, pMETAC was synthesized with atom‐transfer radical polymerization (ATRP) with a similar molecular weight and net charge as selected antimicrobial peptides.^[^ ^45^ ^]^ A weight‐averaged molecular weight (*Mw*) of 3.3 kDa and a number‐averaged molecular weight (*Mn*) of 2.65 kDa (DI = *Mw/Mn* = 1.26) were achieved (see Supporting Information for further information). The composition‐triggered formation of pMETAC/AP205 VLP composites was then studied with SAXS. In our previous study, this polymer triggered the aggregation of Qbeta into colloidal crystals through specific adsorption events.^[^ ^10^ ^]^

**Figure 4:**
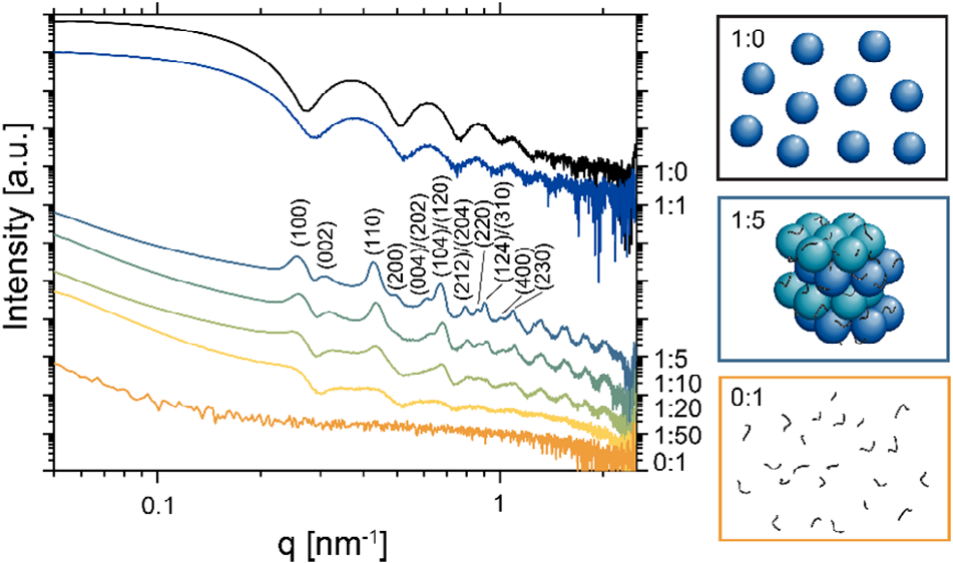
SAXS of AP205 VLPs in PBS at pH 7.4 with increasing pMETAC content (AP205 VLP:pMETAC) and their artistic representation of the sample 1:0, 1:5, and 0:1 as inserts (color coded to the SAXS curves). For the AP205 VLP:pMETAC = 1:5 and 1:10, the Bragg peaks in the SAXS curves are assigned with their Miller indices (*hkl*), indicating a hexagonal closed‐packed (hcp) structure. The curves were vertically shifted for clarity.

In the scattering pattern of AP205 VLP/pMETAC in PBS at weight ratios of 1:0 to 1:1, a shift of the minima to smaller q can be observed (see **Figure** **4**). This indicates a slight shrinkage of the VLP size in the presence of the polycation. Besides, the form factor, *P(q)*, scattering of AP205 VLP is dominating the overall pattern, as seen in Figure 1A and Figure 3. Between 1:5 and 1:20, Bragg reflections and an upturn in the low‐q < 0.2 nm^−1^ appear in the SAXS curves. This upturn arises from attractive interactions among the AP205 VLPs. Together with the Bragg patterns, this demonstrates the formation of internally geometrically ordered AP205 suprastructures. The overall size of these objects is larger than the maximum measurable size with the applied SAXS setup of d = π/q = 78 nm. Upon increasing the pMETAC concentration to 1:50, the Bragg peaks disappear. This likely results from specific adsorptions of pMETAC, leading to charge reversal and repulsive interactions among the polymer coils extending from the “saturated” VLP surface.

The internal structure of the aggregates was further analyzed, based on the position of the Bragg reflections in the SAXS curves. The peaks were indexed with their corresponding *hkl* Miller indices, which match a 3D hexagonal closed‐packed (hcp) structure. The lattice constants, calculated with Equations 6 and 7, are *a* = 28.5 nm and *c* = 40.9 nm. The a‐lattice constant in the hcp structure depicts the distance between the occupied positions in a layer and should correspond to the diameter of the AP205 VLPs. The diameters from the SAXS and DLS reported above are in reasonable agreement with the a‐lattice constant, given the physical difference in the methods and the complexity of defining reflection planes in the core‐shell particle assembly with adsorbed pMETAC.

The aggregation was also performed in VDB to investigate the role of the ionic strength and pH variations in the aggregation process. The SAXS patterns for the AP205/pMETAC 1:5 ratio at pH 7.0 and 8.0 resulted in a hcp structure with comparable dimensions as reported for the PBS conditions above (see Figure 3, blue lines). However, at pH 6.5 (orange line), a reduced intensity of the Bragg peaks is observed, with only the major peaks visible. This indicates reduced long‐range order in the structure. The role of the zeta potential on the AP205 VLP/pMETAC aggregation process in VDB was analyzed to understand the pH dependency. AP205 VLPs show a negative zeta potential of −8.9 ± 0.6 mV at pH 7.0 (see Figure 2). The addition of the cationic pMETAC triggers electrostatic attractions, and at an AP205 VLP/pMETAC weight ratio of 1:5, the magnitude of the zeta potential decreases from |−8.9 ± 0.6| to |−1.1 ± 0.5| mV. As shown above, AP205 VLPs are unstable at pH 6.5, aggregating into a partially ordered structure in the abscence of pMETAC. The observed destabilization of the AP205 VLP/pMETAC superstructure at pH 6.5 coincides with the IEP of AP205 VLPs, supporting the hypothesis that electrostatic interactions play a key role in this process.

The differences in the measured zeta potential values between PBS and VDB is mainly due to the higher ionic strength in PBS. This results in a smaller change in the zeta potential upon pMETAC addition to the AP205 VLPs (see Figure 2). The conductivity measurements also reflect the higher ionic strength in PBS (see Figure 2). However, in VDB, at 1:5, the zeta potential is close to 0 mV. The reduction of the zeta potential magnitude upon increasing the pMETAC content relative to AP205 VLPs demonstrates the adsorption of the pMETAC onto the surface of the AP205 VLPs. The resulting hcp packing geometry suggests that pMETAC adsorbs relatively isotopically onto the AP205 VLP.

Despite AP205 VLP and Qbeta being icosahedral shapes, they pack into different colloidal crystals with pMETAC. Qbeta packs into 2D hexagonal structures at Qbeta:pMETAC weight ratio of 1:50.^[^ ^10^ ^]^ AP205 VLP self‐assembles into 3D hexagonal structures with pMETAC of similar size, already at lower pMETAC content. The difference in packing is likely due to the functional group distributions on the capsid surface and potentially also the influence of the RNA on the surface charge.^[^ ^42^, ^46^ ^]^ The magnitude of the zeta potential of AP205 VLPs of |−8.9 ± 0.6| mV in VDB at pH 7.0 is lower than Qbeta, with |−21.4 ± 1.3| mV in the same buffer.^[^ ^10^ ^]^ The difference in zeta potential correlates with the lower quantity of pMETAC needed to reduce the electrostatic repulsions to trigger suprastructure formation with the AP205 VLPs.

**Figure 5:**
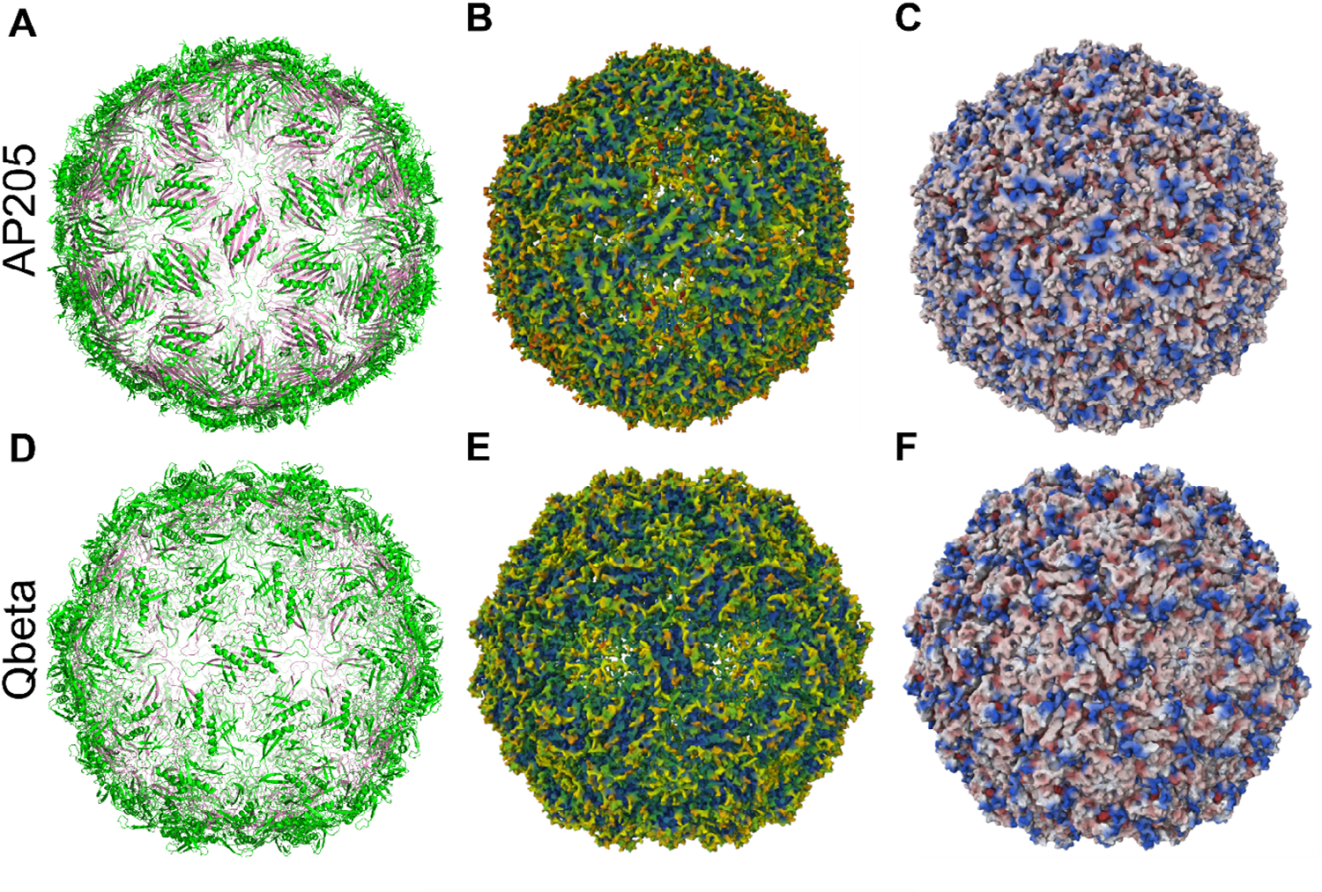
Structural representations of AP205 (PDB ID: 5LQP) (A–C) and Qbeta (PDB ID: 5VLY) (D–F). A) and (D) shows the secondary structure of the peptides in the capsid, whereby the peptides exposed to the internal surface are colored in pink and the peptides exposed to the external surface in green. For (B) and (E), the solvent‐exposed surface, probe radius 1.4 Å, is shown with the relative accessible surface (blue <green< yellow < red). C) and (F) are also solvent‐exposed surface representations with the hydrophobicity at each residue (blue: hydrophilic, red: hydrophobic).^[^ ^54^ ^]^

The surface roughness of AP205 VLP and Qbeta was estimated by analyzing the solvent‐accessible surface area (SASA) from the crystal structures of the protein capsids.^[^ ^47^ ^]^ The ratio of SASA to the theoretical surface area of a smooth sphere of similar radius was used to calculate the roughness factor (RF). This estimation showed that the AP205 VLP with an RF of 5.5 is smoother than the Qbeta with an RF of 6.4 (see Table S2, Supporting Information). This is in agreement with the literature, and it is visualized in **Figure** **5**A,D.^[^ ^41^, ^42^ ^]^ The roughness can also be visualized by the relative solvent‐exposed surface representation of the capsids, where buried parts are in blue‐green and exposed surfaces are in orange to red colors (Figure 5B,E). For the AP205 VLP, more orange and red colored parts of the capsid are visible, and they are also relatively homogenously distributed on the surface. Compared to Qbeta, the relative exposure is reduced with a more inhomogeneous distribution. Additionally, the polarity can be visualized for the individual amino acids, showing that the hydrophilic residues are more homogeneously distributed on the AP205 VLP than the Qbeta surface (see Figure 5C,F).

The hcp packing of the AP205 VLPs is likely a result of a relatively homogeneous distribution of the charged functional groups and its relatively smooth surface, allowing relatively isotropic interactions between the individual particles.^[^ ^41^, ^42^ ^]^ In contrast, Qbeta, with a more irregular surface and a more “patchy” charge distribution, packs into a 2D hexagonal structure, which requires more anisotropic inter‐particle interactions. Those anisotropic interactions have been investigated with Janus‐like particles or other colloidal patchy particles, which have been shown to form various structures.^[^ ^48^, ^49^, ^50^, ^51^ ^]^ Complex ordering, such as quasicrystals, can be explained by the principle of patchy surfaces, with varying numbers of patches and directional bonds of different strengths.^[^ ^52^ ^]^ Therefore, it is likely that the patchiness with charged and hydrophobic areas on the nanocages can induce directional interactions. It appears that electrostatic interactions dominate in the charged surface regions during aggregation, whereas van der Waals, and other interactions contribute in a seconary refinement step. Electrostatic interactions likely govern the initial interaction due to their longer range influence, decaying with distance as 1/r^2^ ‐1/r^4^, while dispersion forces become more significant at shorter distances as they decay with ≈1/r^6^.^[^ ^53^ ^]^

A common feature of the AP205 VLPs and Qbeta‐based colloidal crystals is their stability and responsiveness to pH and ionic strength, making them interesting candidates for various biomedical applications, such as antigen delivery. Cellular uptake of particles is roughly inversely proportional to their size, whereby there is commonly a sweet spot due to the increased binding probability of larger particles or physicochemical properties, such as the faster uptake for softer particles.^[^ ^55^, ^56^, ^57^ ^]^ The stiffness of the investigated colloidal crystals can potentially be tuned by adjusting the surrounding polymer matrix. For the AP205 VLP/pMETAC system, the positive charge of pMETAC and the reduced magnitude of the zeta potential of the aggregates may enhance interaction with negatively charged cell membranes. Similar electrostatic interactions were effective for cell‐penetrating peptide‐modified nanoparticles.^[^ ^58^ ^]^ The delivery of VLPs incorporated in micron‐size particles has been extensively studied, including in vivo, and demonstrated enhanced stability and improved performance from a sustained release.^[^ ^59^, ^60^, ^61^, ^62^ ^]^ Consequently, the AP205 VLP/pMETAC supraparticles can serve as a local and pH‐triggered depot, where individual VLPs are protected inside the supraparticle, and sustainably released over time and deliver their load to surrounding cells. Moreover, the AP205 VLP colloidal crystal system can be adapted by selecting a specific polycation that activates the complement system and enhances the immune response, as shown in another VLP delivery system with microcrystalline tyrosine.^[^ ^63^ ^]^ Altogether, with our proposed system, many parameters can be tailored to increase the functional efficacy of VLPs. Here, the AP205 VLP with cationic polymeric material represents a novel delivery system with the potential to be custom‐tailored for various delivery applications.

## 3 Conclusion

The self‐assembly of AP205 VLPs with a short, 2.65 kDa, polycation pMETAC is highly composition, pH, and ionic strength dependent. At elevated pH above 6.5, and AP205 VLPs/pMETAC ratio for an apparent zeta potential ≈0 mV, 3D hexagonal closed‐packed colloidal crystals were formed. Reducing the pH below 6.5 triggers structural modifications and eventually disaggregation of the colloidal crystals. This pH‐response can guide the creation of functional VLP delivery systems. The comparison to the previously published work on Qbeta‐based colloidal crystals suggests that the roughness of the protein nanocage surface and the directionality of interaction forces play an essential role in this process.^[^ ^10^ ^]^ While these effects have been observed in simulations or simplified Janus particles,^[^ ^48^, ^52^ ^]^ to the best of our knowledge, they have not yet been demonstrated for biologically derived protein nanocages. The short‐chain pMETAC triggered AP205 VLPs and Qbeta aggregation into colloidal crystals, highlighting that this approach is highly versatile for various applications. Qbeta's antimicrobial activity is maintained after self‐assembly, while AP205 VLP could offer a high concentration assembly of vaccine vectors for sustained delivery.^[^ ^10^, ^35^, ^40^ ^]^ Future studies on cargo‐loaded and unloaded nanocages can provide insight into how the cargo loading influences nanocage self‐assembly and how these structures can be further optimized for tailored interaction with mucus and cells for advanced drug and vaccine delivery applications.

## 4 Experimental Section

### Materials

[2‐(methacryloyloxy)ethyl] trimethylammonium chloride solution (METAC) (75 wt % in H_2_O, Sigma–Aldrich), copper (II) bromide (CuBr_2,_ 99% Sigma–Aldrich), Ethyl α‐bromoisobutyrate 98% (EBiB 98%, Sigma–Aldrich), L‐Ascorbic Acid (AscA, 99%, Sigma–Aldrich), 2,2′‐Bipyridyl (BiPy, >99%, Sigma–Aldrich and methanol (MeOH, >99.8%, Fisher Chemicals) were used as is. In all cases, ultra‐pure water (resistivity > 18 MΩ cm) was used. Buffer solutions were made with NaCl (≥99.5% purity, Sigma–Aldrich Chemie GmbH, Steinheim, Germany), KCl (≥99%, Carl Roth, Germany), Na_2_HPO_4_∙2H_2_O (≥98.0% purity, Sigma–Aldrich Chemie GmbH, Steinheim, Germany) KH_2_PO_4_ (>99.5%, Sigma–Aldrich, Germany), NaOH (≥99% purity, Carl Roth GmbH, Karlsruhe, Germany, HCl (ACS reagent grade, Sigma–Aldrich, Buchs, Switzerland), and ultra‐pure water (resistivity > 18MΩ cm).

### Preparation of Buffer solution

Phosphate buffered saline (PBS, 1x) was prepared in ultra‐pure water (resistivity > 18 MΩ cm) containing 137 mM, 2.7 mM KCl, 10.1 mM Na_2_HPO_4_ 2H_2_O, and 1.8 mM KH_2_PO_4_ and was adjusted to pH 7.4 with HCl and NaOH solutions (0.5, 1, and 5 M). The virus dilution buffer (VDB) was prepared by adding 5 mM NaH_2_PO_4_∙2H_2_O and 10 mM NaCl in ultra‐pure water. The pH of the buffer was adjusted to 7.0 using NaOH and HCl.

### Synthesis Poly [2‐(methacryloyloxy)ethyl] Trimethylammonium Chloride (pMETAC)

Details of the synthesis can be found in the previous publication.^[^ ^10^ ^]^ In short, pMETAC was polymerized by atom‐transfer radical polymerization (ATRP) in methanol with CuBr_2_ BiPy, AscA, and EBiB as an initiator. The solution was degassed by three freeze‐thawing cycles, and the reaction was performed under Argon for 18 hrs at 25 °C. The polymer was purified by repeated aceton precipitation, centrifugation, and redissolution in methanol cycles. After the last centrifugation cycle, the pellet was dried in an oven at 40 °C until constant weight. The purified pMETAC was characterized with gel‐permeation chromatography (GPC) as described before.^[^ ^10^ ^]^

### AP205 VLP Production

Recombinant production and purification of AP205 VLPs are reported in detail elsewhere.^[^ ^35^ ^]^ Briefly, recombinant AP205 coat proteins were expressed in *E. coli* BL21‐Gold (DE3, Agilent) cytoplasm. Coat protein dimers self‐assemble into AP205 VLPs in the bacterial cytoplasm.

Bacteria were lysed, DNA and RNA digested, and VLPs were purified via Ni^2+^ mediated precipitation. Glycolipids were extracted, and VLPs were dialyzed against PBS prior to storage at ≈10 mg ml^−1^ in 10% glycerol at −80 °C. The plasmid used for VLP expression is described previously.^[^ ^35^ ^]^ The sequence of AP205 monomer used in this study is MANKPMQPITSTANKIVWSDPTRLSTTFSASLLRQRVKVGIAELNNVSGQYVSVYKRPAPKPEGCADACVIMPNENQSIRTVISGSAENLATLKAEWETHKRNVDTLFASGNAGLGFLDPTAAIVSSDTTAGSGGAHATANATAHHHHHH.

### Dynamic Light Scattering (DLS)

DLS measurements were performed at an incident wavelength of 633 nm at a fixed angle of 173° by averaging 3 runs of 30 s long using Zetasizer Nano (Malvern Panalytical). The autocorrelation function of scattered intensity was analyzed by CUMULANT and CONTIN methods. The VLP concentration was adjusted to 0.1 mg mL^−1^ in filtered PBS and VDB before measurements.

### Small‐Angle X‐Ray Scattering (SAXS)

SAXS measurements were conducted on the SAXSpoint 5.0 (Anton Paar, Graz, Austria) coupled to a MetalJet D2 X‐ray source (Excillum, Kista, Sweden). An X‐ray beam with a wavelength (λ) of 0.134 nm (9.3 keV) and a sample‐to‐detector distance of 1608.1 mm provides a *q*‐range from 0.04 – 2.4 nm^−1^. The scattering vector magnitude *q* was calculated with Equation 1, where n is the refractive index, which is virtually unity for X‐rays in this study, θ is the scattering angle.

(1) $$q=\frac{4\pi n}{\lambda }\;\sin\;\left(\frac{\theta }{2}\right)$$

The scattering and diffraction images were recorded using a 2D EIGER R 1 M detector (Dectris Ltd., Baden, Switzerland) with a total area of 77.10 × 79.65 mm^2^ and pixel size of 75 × 75 µm^2^. The resulting 2D scattering patterns were radially integrated into the 1D *I(q)* functions using SAXSanalysis 4.20 (Anton Paar, Graz, Austria). The temperature was kept at 25 °C if not otherwise mentioned. Measurements were done at a minimum of sextets to check for beam damage, and the average of the results was used. No beam damage was observed. The scattering curves were corrected for transmittance. Buffer scattering was measured with all samples and subtracted as background from the scattering curves.

The SAXS data were analyzed using the generalized indirect Fourier transformation (GIFT) method^[^ ^43^, ^44^ ^]^

The scattering intensity *I(q)* of *N* monodisperse, homogeneous, and spherical particles is described by the product of the form factor *P(q)* and the structure factor *S(q)*:

(2) $$I\left(q\right)=N\;S\left(q\right)P\left(q\right)$$

The GIFT method allows the simultaneous fitting of *P(q)* and *S(q)* by selecting a suitable *S(q)* model.^[^ ^43^, ^64^, ^65^, ^66^ ^]^ The monodispersed charged sphere *S(q)* model with the Yukawa potential and the Hypernetted chain (HNC) closure was used to account for potential inter‐particle scattering effects in this work.^[^ ^43^ ^]^

For the effective charge, the zeta potential was used to approximate the surface potential *ψ_p_* in the following equation (Equation 3), where the charge of the sphere with radius *a* is calculated.^[^ ^67^ ^]^

(3) $$Q_{0}=4\pi \varepsilon_{0}\varepsilon_{r}\psi_{p}a$$

ɛ_*r*_ is the relative permittivity of the buffer at 76.64, *ɛ_0_* is the permittivity of the vacuum of 8.854 × 10^−12^ C V^−1^ m^−1^.

The resulting pair density distribution function *p(r)*, calculated from the *P(q)* contains direct information on the size and shape of the particles.^[^ ^68^, ^69^ ^]^

(4) $$P\left(q\right)=4\pi \;\int_{0}^{\infty }p\left(r\right)\frac{\sin\left(qr\right)}{qr}\;dr$$

where:

(5) $$p\left(r\right)=r^{2}{\tilde{\rho }}^{2}\left(r\right)$$

with ${\tilde{\rho }}^{2}\left(r\right)$ being the convolution square of the spatially averaged excess electron density *ρ(r)*, relative to the buffer. In the case of spherical geometry, deconvolution of the *p(r)* gives the radial contrast profile in electron density *ρ(r)* relative to the mean value of the solvent. This gives information about the internal structure of the scattering particles.^[^ ^70^, ^71^ ^]^ The fitting parameter can be found in Table S1 (Supporting Information).

Bragg peaks in the SAXS curves are the results of the formation of ordered structures. For these samples, the corresponding reflections, defined by their *hkl* Miller indices, were assigned to the space groups of the liquid crystalline structures.

(6) $$d_{hkl}=\frac{2\pi }{q_{hkl}}$$

(7) $$\frac{1}{{d^{2}}_{hkl}}=\frac{4}{3}\frac{\left(h^{2}+hk+k^{2}\right)}{a^{2}}+\frac{l^{2}}{c^{2}}$$

where *q_hkl_* is the *q*‐value of the Bragg peak corresponding to the reflection from the *hkl* Miller planes and *d_hkl_* is the corresponding interplanar spacing. The lattice constants *a* and *c* were calculated from *d_hkl_* using Equations 6 and 7, assuming a hexagonal close‐packed system. This structure provided the best possible fit to all peaks out of all the tested symmetries.

### Electrophoretic Mobility Measurements for Zeta‐Potential and Conductivity

The Litsesizer 500 (Anton Paar, Graz, Austria) was used to determine the electrophoretic mobility, *µ_e_*, by phase analysis of the light scattering by the sample. The wavelength of the laser was 658 nm, the laser power was 40 mW, and the scattering angle was either 15° or 175°, optimized automatically through the built‐in algorithm of the device. The temperature was 25 °C for all measurements. The zeta‐potential, ζ, was calculated with Henry's equation.^[^ ^73^ ^]^

(8) $$\mu_{e}=\frac{2\varepsilon_{r}\varepsilon_{0}\zeta \;f\left(\mathrm{ka}\right)}{3\eta }$$

*ɛ_r_* is the relative permittivity of the buffer at 76.64, *ɛ_0_* is the permittivity of the vacuum of 8.854 × 10^−12^ C V^−1^ m^−1^, f(ka) is the Henry function, approximated as 1.5 under the Smoluchowski approximation,^[^ ^74^ ^]^ and *η* is the viscosity of the buffer (0.904 mPa s). The samples were diluted 20 – 100‐fold and vortexed before being measured five times by applying a voltage of 50 V. At the same time, the current *I* was measured on the same samples at the applied voltage *V*. From this, the conductivity *σ* is calculated (Equation 9), where *L* is the distance between the electrodes and *S* is the surface area.

(9) $$\sigma =\frac{I}{V}\times \frac{L}{S}$$

### Estimation of Surface Roughness of Protein Capsids

The surface roughness of VLPs was quantified by calculating the solvent‐accessible surface area (SASA), accessible to a solvent molecule of radius 1.4 Å, and other structural parameters calculated from the crystal structures obtained from high‐resolution cryo‐TEM images of VLP structures in the protein data bank PDB (AP205 (PDB ID: 5LQP) and Qbeta (PDB ID: 5VLY)).^[^ ^41^, ^47^, ^75^ ^]^

Roughness Factor (RF) is defined as SASA on the external surface (SASA_ext_) divided by the area of a sphere of radius equal to the capsid external radius (r_ext_) (see Equation 10). A cutoff radius to mark external versus internal surfaces is defined as the mean of the external and internal radii (r_int_) of the capsid. These structural parameters were extracted using PyMOL version 2.5.3.

(10) $$RF=\frac{SASA_{ext}}{4\pi \;r_{ext}^{2}}$$

## Conflict of Interest

The authors declare no conflict of interest.

## Supporting information



Supporting Information

## Data Availability

The data that support the findings of this study are available in the supplementary material of this article.
