# Supplementary material for: Colloidal Crystallization of Virus‐Like Particles with Polycations
Source: Small. 2025 Jul 2;21(34):2503579. doi: 10.1002/smll.202503579 (PMC12393024; doi:10.1002/smll.202503579)
Supplement: Supplementary file 1 — Supporting Information [file SMLL-21-2503579-s001.docx]

**Supporting Information**

**Colloidal Crystallization of Virus-Like Particles with Polycations**

*Bettina Tran^1^, Timothy G. Keys,^2^ Milad Radiom^2*^, Stefan Salentinig^1*^*

^1^ Department of Chemistry, Food Research and Innovation Center and National Center of Competence in Research Bio-inspired Materials, University of Fribourg, Chemin du Musée 9, 1700 Fribourg, Switzerland

^2^ Department of Health Sciences and Technology, Eidgenössische Technische Hochschule Zürich, Schmelzbergstrasse 9, 8092 Zürich, Switzerland

## **Supporting Figures**


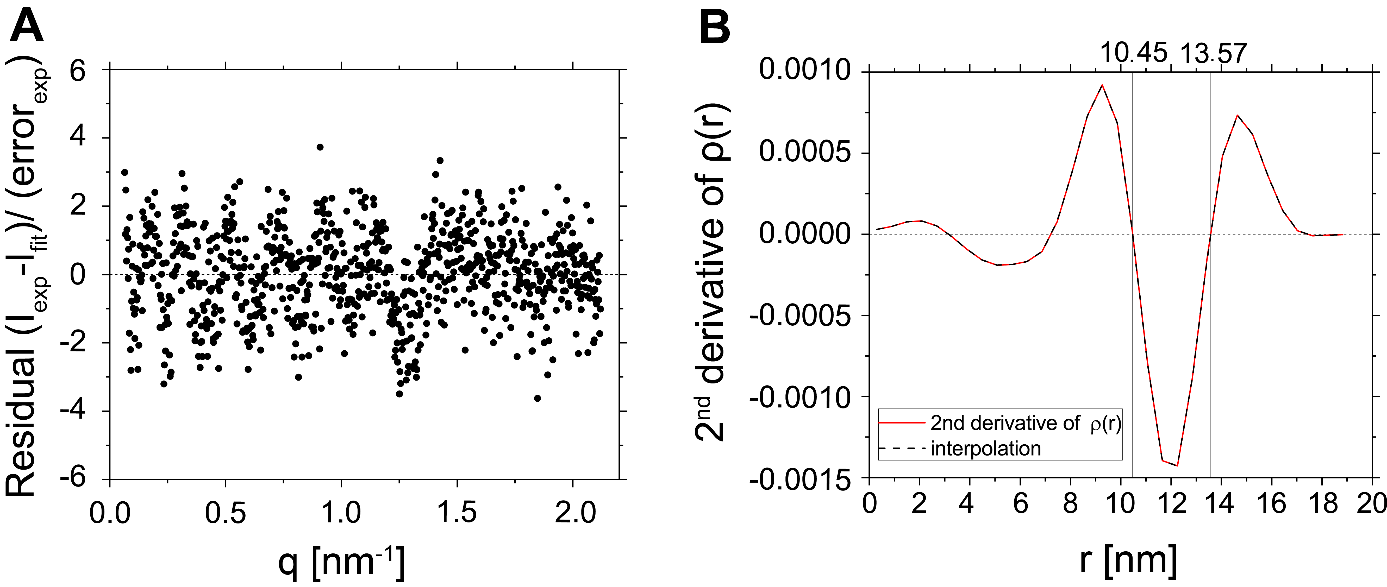


Figure S1: (A) Residuals from the GIFT fitting of 1wt% AP205 VLP in PBS pH 7.4. (B) 2^nd^ derivative of electron density distribution ρ(r) from GIFT fitting (red) and its interpolation (black dashed) to find the point of inflections in the ρ(r), for 9 < r < 16 nm.


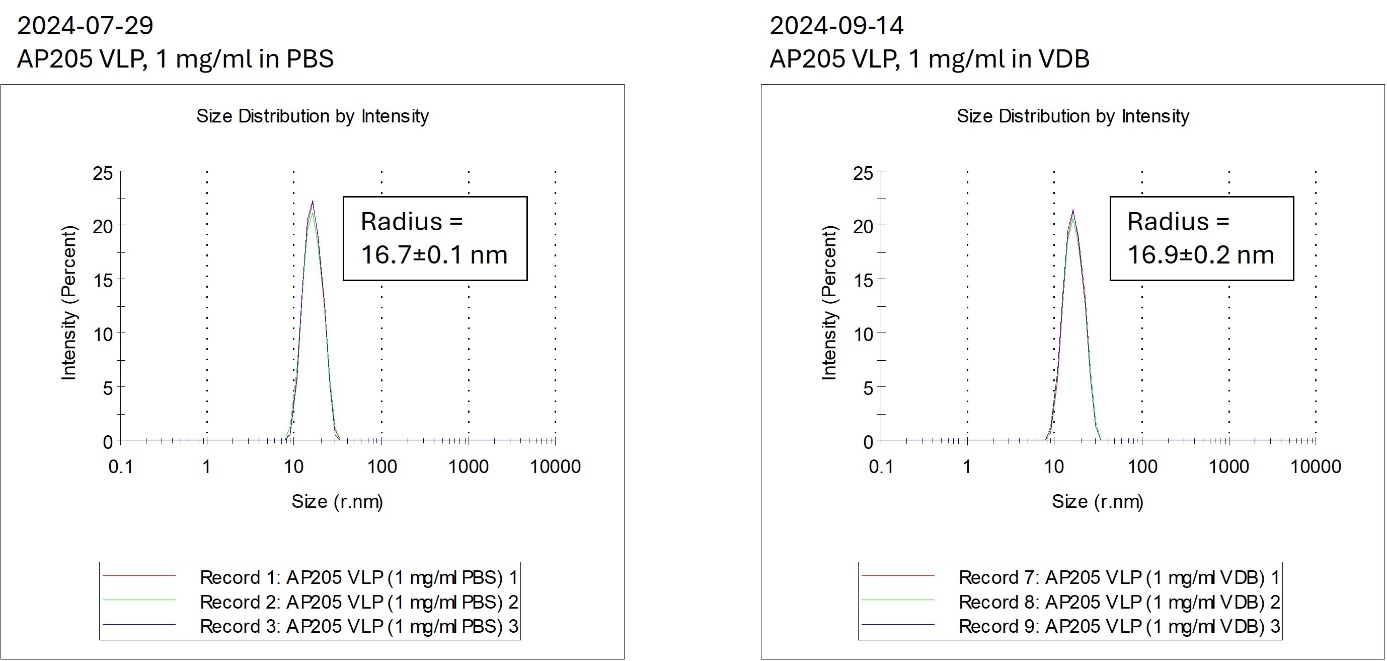


Figure S2: Comparison of the size of AP205 VLP in PBS versus VDB measured in DLS. The correlation functions are presented in Figure S3.


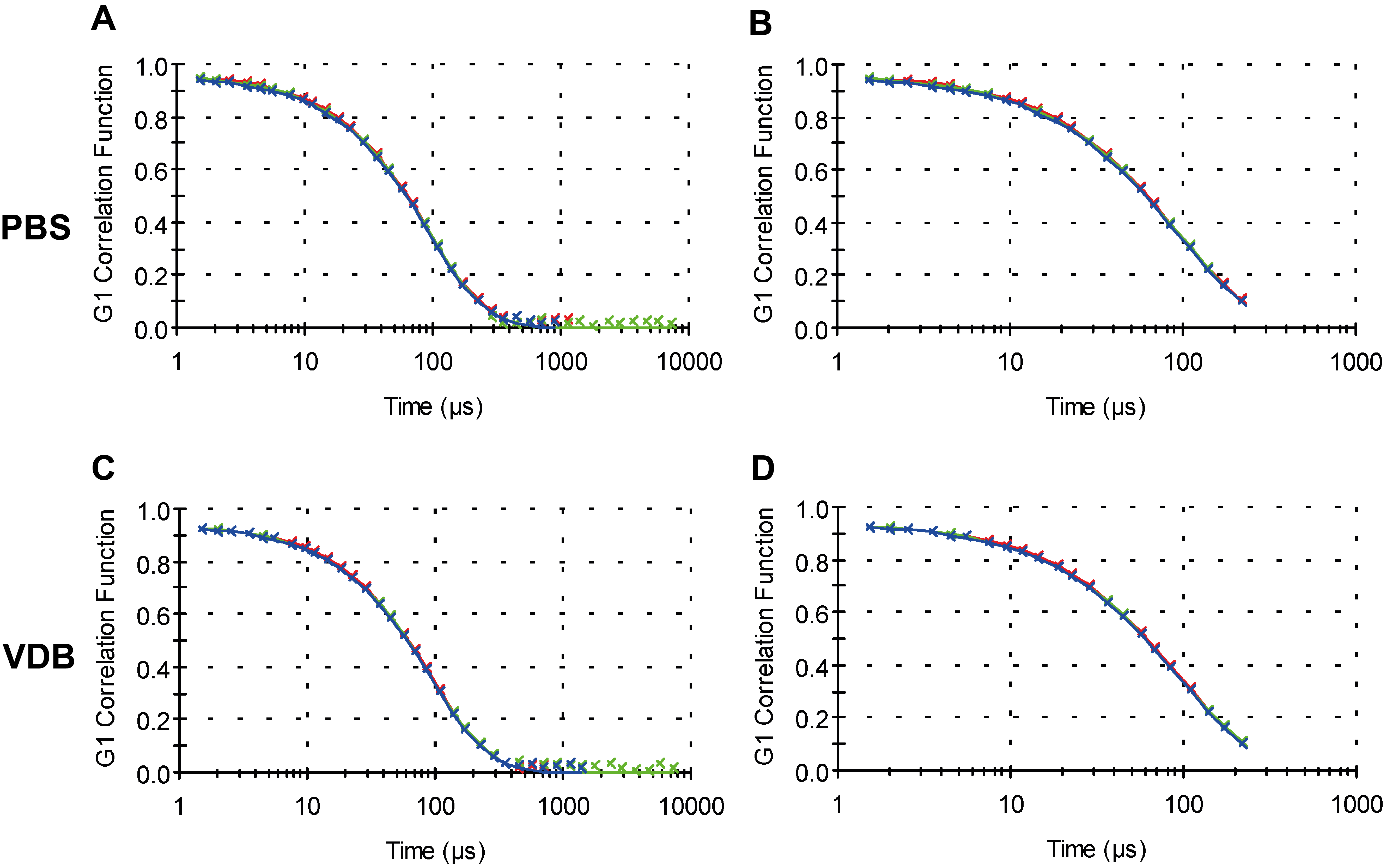


Figure S3: Correlation functions to the DLS of AP205 VLP in PBS pH 7.4 with the Contin (A) and cumulant (B) analysis (Z-ave = 15.8 and PDI = 0.04), as well as for AP205 VLP in VDB pH 7.0 with the Contin (C) and cumulant (D) analysis (Z-ave = 15.9 and PDI = 0.05).

Figure S4: SAXS pattern of AP205 VLP: pMETAC at 1:0 and 1:5 weight ratio in PBS (solid lines) overlayed with the sample in VDB (lighter colored, dashed lines). The curves for 1:0 and 1:5 were vertically shifted for clarity.


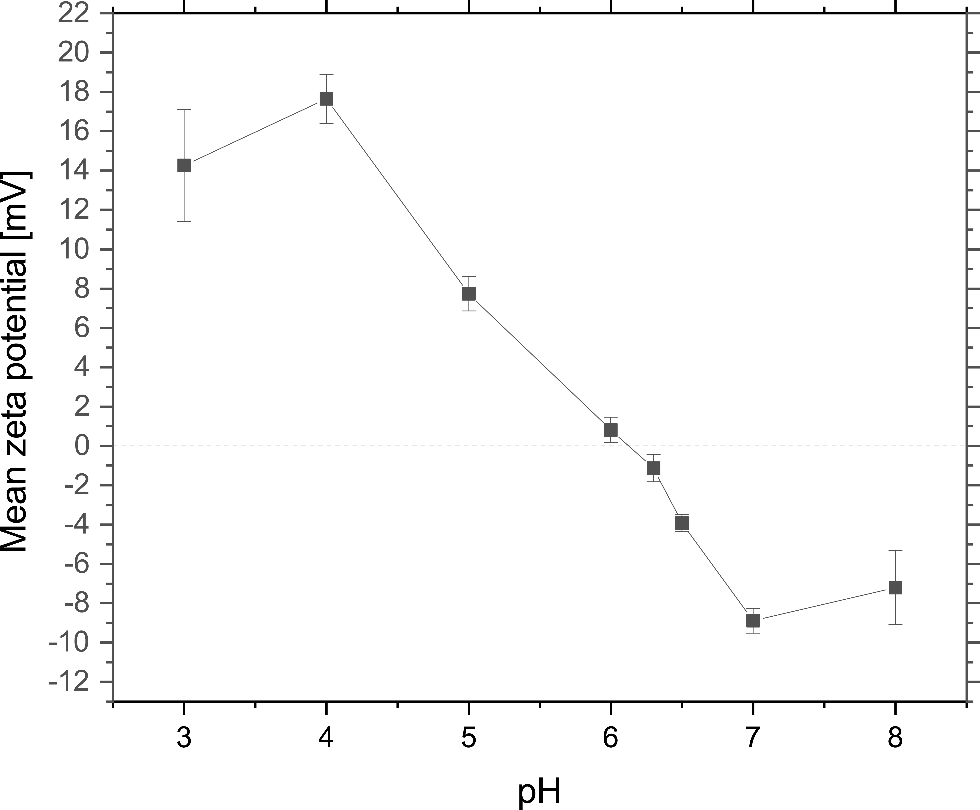


Figure S5: Zeta potential (ZP) of AP205 VLP in VDB showing a negative ZP at pH > 6.3 and an inversion to positive ZP at pH ≤ 6.0. The isoelectric point (IEP) is between 6.0 and 6.3.

## **Supporting Tables**

Table S1: Structure factor parameters for AP205 in PBS, using a charged sphere model. The effective charge of a sphere is calculated from the zeta potential. The salt concentration is calculated for monovalent ions. See materials and methods for details.

| **Sample** | **1wt% AP205 VLP in PBS** |
| --- | --- |
| Volume fraction | 0.0115 ± 0.0007 |
| R_app_ [nm] | 18.3 ± 0.5 |
| Effective charge | -8.1 (fixed) |
| Salt concentration [mol/L] | 0.162 (fixed) |
| Dielectric constant | 78.3 (fixed) |
| Temperature [K] | 298 (fixed) |

Table S2: Roughness factor (RF) calculated from the parameters extracted from the crystallographic data in the protein data bank (PDB) and the analysis of the solvent accessible surface area (SASA), with a probe of 1.4Å. Roughness Factor (RF) is calculated from the SASA on the external surface (SASA_ext_) divided by the area of a sphere of radius equal to the capsid external radius (r_ext_). See materials and methods for details.

| **VLP**  **(PDB ID)** | **r_ext_**  **[Å]** | **r_int_ [Å]** | **Cutoff Radius [Å]** | **SASA_ext_**  **[Å²]** | **RF**  **[-]** |
| --- | --- | --- | --- | --- | --- |
| AP205 (5lqp) | 145.14 | 114.00 | 129.57 | 1456681.4 | 5.5 |
| Qbeta (5vly) | 139.58 | 102.88 | 121.23 | 1566926.4 | 6.4 |
